# A Novel *VPS13A* Deletion in *VPS13A* Disease (Chorea-Acanthocytosis): A Case Report with Brief Literature Summary

**DOI:** 10.3390/ijms262311521

**Published:** 2025-11-27

**Authors:** Benedetta Perrone, Viviana Mosca, Martina Pecoraro, Paola Ruffo, Elda Del Giudice, Alberta Leon, Martina Maino, Vincenzo La Bella, Rossella Spataro, Francesca Luisa Conforti

**Affiliations:** 1 Laboratory of Medical Genetics, Department of Pharmacy and Health and Nutritional Sciences, University of Calabria, Rende, 87036 Cosenza, Italy; benedetta.perrone@unical.it (B.P.); paola.ruffo@unical.it (P.R.); 2 Health Center, Department of Pharmacy and Health and Nutritional Sciences, University of Calabria, Rende, 87036 Cosenza, Italy; 3 Lab of Neurochemistry, Department of Biomedicine, Neurosciences, and Advanced Diagnostics (Bi.N.D.), University of Palermo, 90127 Palermo, Italy; vivianamosc@gmail.com (V.M.); martina.pecoraro.mp@gmail.com (M.P.); vincenzo.labella@unipa.it (V.L.B.); 4 R&I Genetics Srl, 35127 Padua, Italy; edelgiudice@rigenetics.com (E.D.G.); aleon@rigenetics.com (A.L.); mmaino@rigenetics.com (M.M.); 5 Intensive Neurorehabilitation Unit, Villa delle Ginestre Hospital, ASP Palermo, 90141 Palermo, Italy; spatarorossella@gmail.com

**Keywords:** Chorea-acanthocytosis, *VPS13A*, VPS13A, deletion, next-generation sequencing, fibroblasts

## Abstract

*VPS13A* disease is a rare, autosomal-recessive, neurodegenerative disorder characterized by involuntary movements, orofacial dystonia, seizures, psychiatric symptoms, and the presence of spiky, deformed red blood cells (acanthocytes). The disease is caused by mutations in the *VPS13A* gene, which encodes the VPS13A protein (previously known as chorein). This protein is a member of the family of bridge-like lipid transport proteins, involved in bulk lipid transfer between membranes and intracellular vesicle trafficking. We describe the case of a 37-year-old woman with gait instability, semi-flexed legs, and involuntary distal muscle movements. Genetic testing was performed using next-generation sequencing (NGS), followed by molecular analysis. Fibroblasts from the patient, her mother, and a healthy control were analyzed by immunofluorescence and Western blotting. NGS identified a novel homozygous 2.8 kb deletion encompassing exons 69–70 (69–70del) of the *VPS13A* gene (NM_033305.3). The same variant was detected in the patient’s mother in a heterozygous state and her brother in a homozygous state. Although other deletions in the gene have been described, a comprehensive search of population variant databases and the existing literature did not reveal previous reports of this deletion. Fibroblasts from the patient, her mother and a healthy control were characterized. Functional assays showed a complete absence of the VPS13A protein in the patient’s fibroblasts. This study expands the mutational spectrum of *VPS13A*-linked *VPS13A* disease and underlines the importance of comprehensive genetic analysis in atypical cases.

## 1. Introduction

*VPS13A (Vacuolar protein sorting 13 homolog A)* disease (previously known as Chorea-acanthocytosis, ChAc; OMIM #200150) [1], is the most common subtype of neuroacanthocytosis syndrome, characterized by the presence of abnormal star-shaped red blood cells (acanthocytes) and neurological disturbances [2]. First identified in Japan, where subsequent cases have also been described, *VPS13A* disease is now recognized worldwide [3,4], although it has an extremely low overall prevalence of fewer than 1000 reported cases (https://www.orpha.net/it/disease/detail/263440 accessed on 15 July 2025). Clinically, *VPS13A* is characterized by involuntary movements and dystonia of the limbs and oro-facial muscles as well as the presence of acanthocytes (acanthocytosis) [5]. Hearing may also be impaired, as contractions of the tongue and throat can interfere with chewing and swallowing food [6]. There have also been cases where cognitive and psychiatric disorders have preceded the first neurological manifestations. Finally, seizures are the first manifestation of the disease in at least one-third of patients [7]. The age of onset is usually between 20 and 50 years, with a mean of around 30–35 years, and an average diagnostic delay of ~6 years [8]. Despite growing awareness, the causative mechanism of the pathology is still unknown, and *VPS13A* disease remains underdiagnosed due to its phenotypic overlap with other neurodegenerative and psychiatric disorders [5]. Therefore, a definitive diagnosis cannot be made based on instrumental tests and the morphological study of acanthocytes alone. In this context, genetics plays a crucial role in identifying the molecular cause of the disease. *VPS13A* disease is caused by biallelic mutations (autosomal recessive transmission) in the *VPS13A* gene, located on chromosome 9 (9q21) and spanning 250 kb [9]. This gene (NM_033305.3) comprises 72 exons and encodes a 3174-amino acid protein VPS13A (360 kDa). This protein belongs to the recently characterized family of bridge-like lipid transfer proteins (BLTPs), which mediate non-vesicular bulk lipid transport between closely apposed membranes at organelle contact sites [9,10,11]. The VPS13A protein has a complex domain structure that includes an N-terminal domain, which contains an FFAT (two phenylalanines in an acidic tract) motif for binding to the endoplasmic reticulum (ER) vesicle-associated membrane protein (VAMP)-associated protein (VAP). It also has a VAB (VPS13 Adaptor Binding) domain and a CHOREIN domain, which likely function in lipid transfer, an APT1 domain (aberrant pollen transcription 1), a C-terminal ATG2 domain (ATG_C), and a PH-like domain (Pleckstrin homology-like domain) located more towards the C-terminal. These domains allow VPS13A to associate with various organelles, including the ER, mitochondria, endosomes, and lipid droplets, facilitating lipid transfer and membrane contact sites (MCSs) [12,13,14].

Recent studies have further explored the pathophysiological mechanisms and potential biomarkers of *VPS13A* disease, providing insights into how mutations in this large lipid transport protein lead to neurodegeneration [9]. Several mutations in the *VPS13A* gene have been identified to date, including gross deletions (*n* = 8), nonsense and missense mutations (*n* = 64), and splicing mutations (*n* = 34), as reported in the Human Gene Mutation Database (HGMD; https://www.hgmd.cf.ac.uk/ac/index.php accessed on 2 October 2025).

Supplementary Table S1 provides a comprehensive list of the 92 *VPS13A* variants that have been identified as either pathogenic or likely pathogenic, and which are associated with *VPS13A* disease in populations worldwide [15,16,17,18,19,20,21,22,23,24,25,26,27,28,29,30,31,32,33,34,35,36]. The majority of these (65%) are nonsense or frameshift mutations predicted to lead to a truncated protein. Splice-site variants represent 20% of the total, while large deletions/duplications and missense substitutions account for around 8% and 5%, respectively. These variants are distributed throughout the *VPS13A* coding sequence, with no single major mutational hotspot [26]. In this report, we describe the first detailed characterization of the functional properties of the occurring deletions of exons 69 and 70 in *VPS13A* (*VPS13A* 69-70del; NM_033305.3; chr9:77403236-77405987; c.9190_9419del; p.V3064_K3133del) in a *VPS13A* disease patient, expanding the known mutational spectrum and emphasizing the importance of combining genetic, cellular, and clinical data for accurate diagnosis.

## 2. Case Description

The mother of the proband reported she had a complex congenital heart disease at an early age, including Botallo ductus patency, for which she underwent unspecified surgery. At 20, she started complaining of early fatigue, which gradually worsened in the following years. At 30, she began experiencing involuntary limb movements, initially affecting only her legs and then gradually spreading to her arms. She then performed a diagnostic pathway, which revealed creatine phosphokinase above the normal range (>2000 UI) and electromyographic signs of polyneuropathy. A biopsy of the left bicipital muscle showed no abnormalities. At 34, family members started observing behavioral changes, with irritability, inflexibility, and obsessive thoughts. She started off-label treatment with tetrabenazine 100 mg/day per os.

The whole clinical condition gradually worsened in the following years, with increasing choreic movements and psychiatric disturbances, which affected the quality of night rest and autonomy as a whole. The patient is now 37 years old and, as a consequence of the gradual clinical worsening, has unstable walking and needs help in most daily activities.

**Figure 1:**
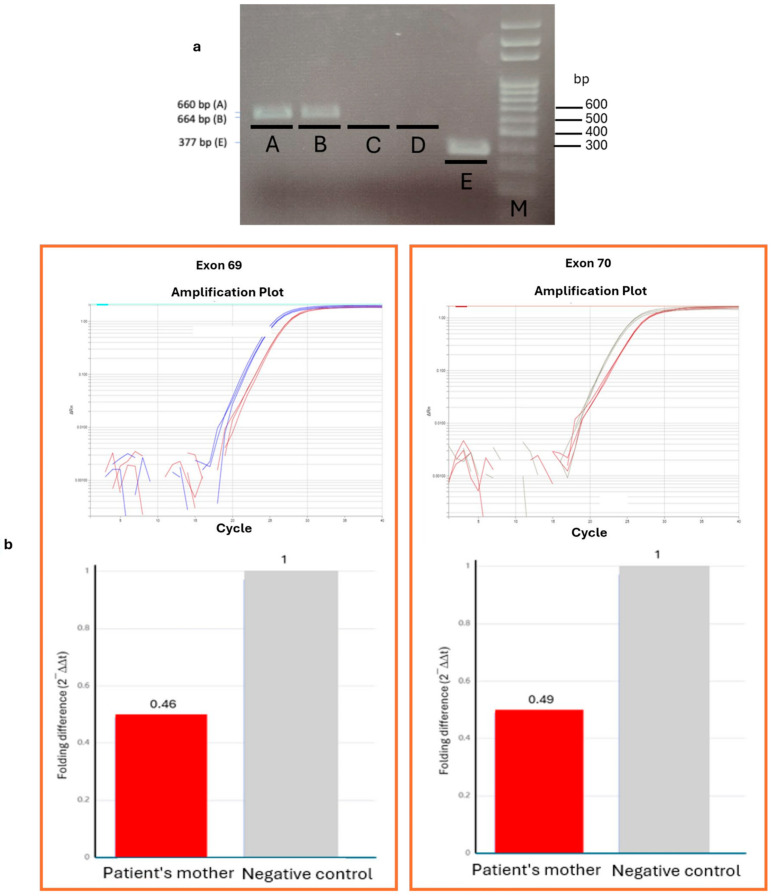
Gel electrophoresis for exons 69-70 of the *VPS13A* gene in the proband and qPCR analysis in the proband’s mother. (**a**) Gel electrophoresis of PCR products for *VPS13A* exons 69-70. Lane A: Negative control for *VPS13A*-exon 69 (652 bp); Lane B: Negative control for *VPS13A*-exon 70 (664 bp); Lane C: PCR product from the proband (P) for *VPS13A*-exon 69; Lane D: PCR product from the proband (P) for *VPS13A*-exon 70; Lane E: Amplification control (337 bp). M: 100 bp DNA ladder. (**b**) Real-time qPCR analysis of *VPS13A* exons 69 (**left**) and 70 (**right**). The red curve corresponds to the mother’s sample, while the blue and grey curves represent the negative controls for exons 69 and 70, respectively. The bottom panels show the corresponding relative quantification histograms (ΔΔCq).

Next-generation sequencing (NGS) analysis was performed using a targeted gene panel comprising the coding regions of 4 genes (Microsomal Triglyceride Transfer Protein (*MTTP),* Pantothenate Kinase 2 *(PANK2), VPS13A* and X-Linked Kx Blood Group Antigen, Kell And VPS13A Binding Protein *(XK*)). Interestingly, a homozygous deletion (69-70del) of 2.,8 Kb in *VPS13A* (NM_033305.3; chr9:77403236-77405987; c.9190_9419del, p.V3064_K3133del) was revealed. This results in the loss of exons 69 and 70 in frame, corresponding to the removal of 70 amino acids. This *VPS13A* mutation has not been reported before and, according to the American College of Medical Genetics and Genomics (ACMG) guidelines [37] for copy number variants (CNVs), it is classified as likely pathogenic, fulfilling criteria 1A (contains protein-coding exons), 2A (involves a known loss-of-function (LoF)-sensitive gene) and 2E (both breakpoints are within the same gene, confirming a gene-level LoF event). The deletion was confirmed in the proband by PCR and gel electrophoresis analysis (Figure 1a). Semi-quantitative analysis performed using real-time polymerase chain reaction (PCR) revealed the presence of the same deletion in a heterozygous state in the proband’s mother (Figure 1b).

**Figure 2:**
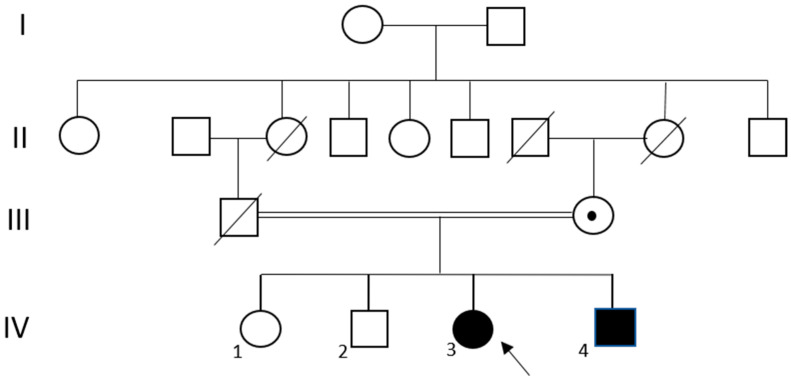
Pedigree of the *VPS13A* disease-affected proband. The proband (IV-3) is indicated by an arrow. The double line between the parents indicates consanguinity. A dot symbol (•) denotes carrier status for the *VPS13A*-related mutation. Black symbols represent affected individuals. Roman numerals indicate generations and Arabic numbers (1,2,4) indicate individuals.

The same 69-70del of the *VPS13A* gene was also detected in the proband’s brother, who developed clinical signs associated with *VPS13A* disease. It is also worth noting that the patient’s parents are first cousins, contributing to the homozygous inheritance of the variant. The family pedigree is shown in Figure 2.

To investigate the functional effects of this novel *VPS13A* variant, a skin biopsy was performed on the patient, as previously described [38]. Specifically, a skin biopsy, immediately preserved in cold phosphate-buffered saline (PBS), was cultured in a complete medium consisting of DMEM (Dulbecco’s modified Eagle’s medium) supplemented with 10% calf serum (CS), 2 mM L-glutamine, 1 mM sodium pyruvate, 100 U/mL penicillin, and 100 U/mL streptomycin. The plate was then placed in a 5% CO_2_ incubator at 37 °C. The medium was changed every three to four days until the released fibroblasts reached 80% confluence (approximately 20 days). All experiments were performed using cells from the third or fourth passage.

**Figure 3:**
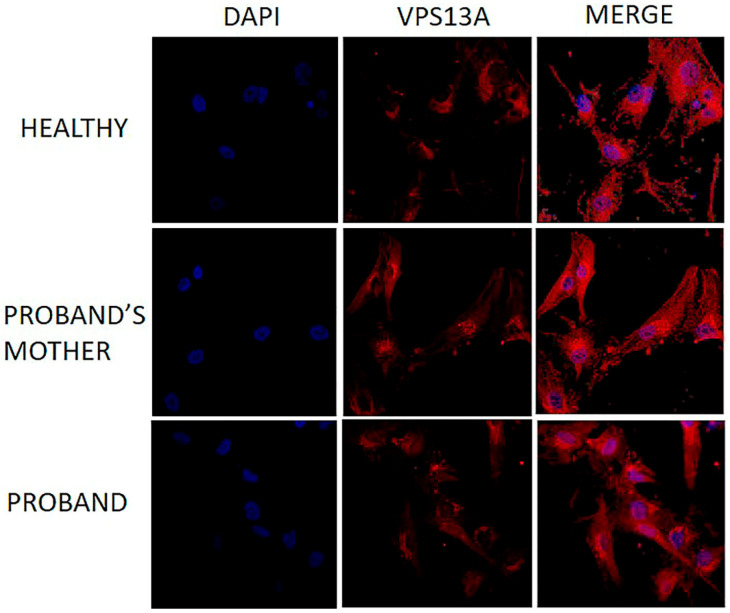
Immunofluorescence analysis of VPS13A protein localisation in fibroblasts. Fibroblasts from a healthy control, a heterozygous carrier (mother), and the homozygous patient (69-70del). Cells were immunostained with a polyclonal anti-VPS13A antibody (red) (Proteintech; 1:500 dilution). DAPI (blue) was used for nuclear staining. In all three cell lines, VPS13A shows a perinuclear localization pattern.

The immunofluorescence (IF) assay on fibroblasts was performed according to protocols that had been published already [39] to assess the subcellular localization of VPS13A, which is mostly perinuclear, as shown in Figure 3. Briefly, fibroblasts were cultured on glass coverslips in a 4-well plate at a concentration of 10,000 cells/mL with two replicates for each subject. The cells were fixed with 4% paraformaldehyde for 30 min at room temperature, then permeabilized with 0.5% PBS-Triton X-100 for 10 min. They were then incubated with a polyclonal VPS13A antibody diluted 1:500 (Proteintech Group Inc., Chicago, IL, USA; catalog number 28618-1-AP).

In addition, to evaluate the level of the VPS13A protein, a Western blot (WB) assay was performed in whole lysate extract. The blots were incubated with a dilution of 1:800 of the polyclonal VPS13A antibody (Proteintech Group Inc., Chicago, IL, USA; catalog number 28618-1-AP) and a dilution of 1:1250 of the monoclonal alpha Tubulin antibody (Sigma-Aldrich—St. Louis, MO, USA; catalog number T6199). Protein bands were visualized using ECL Star (Euroclone—Pero, MI, Italy), and chemiluminescence detection was performed using a ChemiDoc-It Imaging System (UVP, Cambridge, UK).

**Figure 4:**
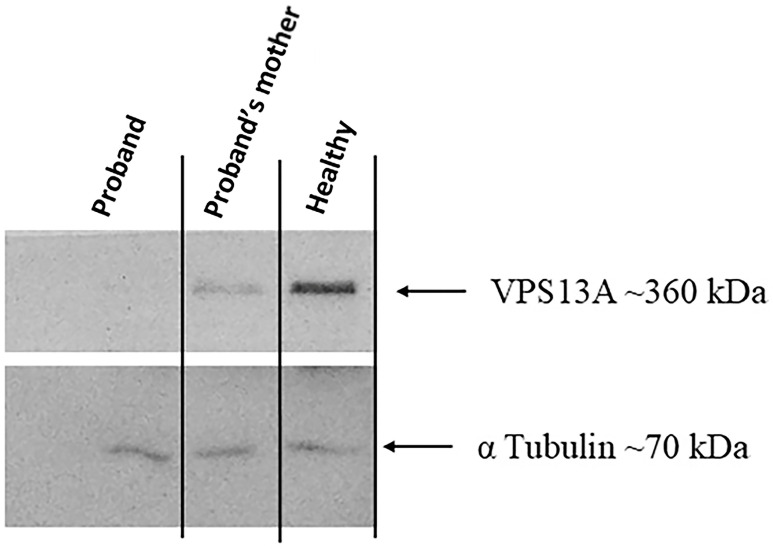
Western blot analysis of VPS13A protein in fibroblasts. WB was performed using an anti-VPS13A antibody recognizing an epitope between amino acids 2452–2595 (dilution 1:800). The predicted molecular weights are approximately 360 kDa for the full-length wild-type (WT) protein and 352 kDa for the putative truncated protein (p.V3064_K3133del) resulting from 69 to 70del. WB detects the expression of the VPS13A protein in a patient with 69-70del in the homozygous state, her mother, who is a carrier of the same mutation in the heterozygous state, and a healthy control. Alpha tubulin (monoclonal antibody; dilution 1:1250—Sigma-Aldrich) was used as a loading control to normalize the levels of protein detected. Biological replicates: *n* = 3 independent experiments. Protein marker: Sharpmass VI (Euroclone). The reference molecular weights used were 150 kDa and 250 kDa.

The patient with *VPS13A* disease lacks the band that marks VPS13A, indicating a loss of protein likely due to the deletion. This is in contrast to her mother and a healthy control, where the band is clearly visible (Figure 4).

Despite positive immunofluorescence staining in the patient’s fibroblasts, VPS13A was not detectable by WB analysis. This finding is explained by the use of the same C-terminal antibody, which recognizes the epitope aa 2452–2595 and can detect trace amounts of an abnormal VPS13A protein present at levels too low for WB detection.

Extended functional assays in fibroblasts were not performed, as it was beyond the scope of this study.

## 3. Discussion

This study reports the case of a 37-year-old female patient whose clinical phenotype—characterized by involuntary limb movements, psychiatric symptoms, polyneuropathy signs on electromyography (EMG), and elevated creatine phosphokinase (CK)—is consistent with previously reported manifestations of *VPS13A* disease. Genetic testing using targeted NGS revealed a previously unreported 2.8 Kb deletion (chr9:77403236-77405987; c.9190_9419del, p.V3064_K3133del) in the *VPS13A* gene, encompassing exons 69 and 70.

To date, approximately 90 pathogenic variants in the *VPS13A* gene have been linked to the *VPS13A* disease phenotype (see Supplementary Table S1). Among these, eight have been reported in Italian patients, including nonsense mutations, frameshift deletions/insertions, and intronic variants [20,22,27].

Overall, eight multiexon deletions (in-frame and frameshift) in the *VPS13A* gene are reported in the HGMD database. However, only five of these have been definitively classified as pathogenic (see Supplementary Table S1), and most of these have only been described at the genomic level. In particular, the deletion of exons 60–61 appears common in the Japanese population [3,16], and the deletion of exons 70–72 has been observed in the French–Canadian population [4]; therefore, the proportion of pathogenic variants detected varies by population. The majority of disease-associated variants of *VPS13A* are predicted to be loss-of-function.

Multiple lines of evidence support the pathogenicity of the exons 69 and 70 in-frame deletion reported in this study: (i) the deleted region encodes a critical portion of the C-terminal domain of VPS13A, including the PH-like domain encoded by the final exons of *VPS13A.* This region is essential for lipid transport and membrane tethering at organelle contact sites (e.g., ER–mitochondria sites). The C-terminal of VPS13A harbors conserved lipid-binding domains (similar to ATG2, the autophagy protein) and interaction motifs for partners like XK [40]. Notably, many patient mutations have been reported in this domain, which is encoded by the last three exons of the *VPS13A* gene [41] (Supplementary Table S1); (ii) the family pedigree revealed consanguinity between the proband’s parents, and segregation analysis confirmed the presence of a homozygous deletion in two affected family members (IV-3 and IV-4); (iii) functional analysis showed a complete absence of full-length VPS13A in the patient compared to the control and the proband’s mother, as demonstrated by WB. However, the residual signal detected by IF is consistent with the presence of trace amounts of an abnormal VPS13A protein, which is likely degraded and/or non-functional. It is important to note that the antibody used for both WB and IF recognizes an epitope (aa 2452–2595) upstream of the region involved in the deletion (aa 3064–3133). Thus, the positive IF signal most likely represents an unstable, incomplete protein that persists at very low levels, below the WB detection threshold. Although the resulting protein may remain “intact” in terms of structure, the loss of these exons would eliminate a region essential for its function, rendering it non-functional.

Rarely, individuals with *VPS13A* disease may express VPS13A that lacks the PH domain, involved in lipid transfer and MCS [41]. In addition, some pathogenic missense substitutions that do not result in misfolding and protein degradation or small deletions leading to expression of a truncated protein are known to be associated with normal levels of VPS13A [41]. Together, our findings support a loss-of-function mechanism, in line with previous studies [15,26,40].

Additionally, the *GnomAD* browser shows a low rate of missense variants in the exons of *VPS13A* and classifies this as a gene with a pLI score of 0.7 (https://gnomad.broadinstitute.org/gene/ENSG00000197969?dataset=gnomad_r4, accessed on 7 July 2025), which indicates that it is likely to be intolerant to loss-of-function variants. This is in line with the severe neurological consequences observed in patients with *VPS13A* disease resulting from *VPS13A* mutations [29].

A clinical comparison with previously described Italian patients, as shown in Supplementary Table S1, reveals an overlap of features, particularly concerning movement disorders, psychiatric symptoms and elevated CK levels. However, the presence of polyneuropathy signs and evidence of cardiac involvement in our patient appear to be less frequently reported findings in Italian patients [20,27].

Taken together, these data further support that the mutation causes VPS13A deficiency and disrupts intracellular lipid trafficking, resulting in the neurodegenerative features of *VPS13A* disease. Our results also underline the importance of combining genetic diagnosis and molecular characterization to correctly classify variants, in line with the ACMG guidelines [37].

## 4. Conclusions

The identification of a novel homozygous *VPS13A* 69-70del highlights the diagnostic importance of NGS in revealing pathogenic variants that elude conventional sequencing approaches. This finding broadens the mutational spectrum of *VPS13A* disease and improves our understanding of its molecular pathology. An early and accurate genetic diagnosis of *VPS13A* disease is crucial, as it facilitates appropriate patient management and genetic counseling and lays the groundwork for future therapeutic approaches in *VPS13A* disease.

## Data Availability

The original contributions presented in this study are included in the article/Supplementary Materials. Further inquiries can be directed to the corresponding author(s).

## Supplementary Materials

The following supporting information can be downloaded at: https://www.mdpi.com/article/10.3390/ijms262311521/s1.

## Abbreviations

The following abbreviations are used in this manuscript:

ACMG	American College of Medical Genetics and Genomics
APT1	Aberrant pollen transcription 1
ATG_C	C-terminal ATG2 domain
BLTPs	Bridge-like lipid transfer proteins
ChAc	Chorea-acanthocytosis
CK	Creatine phosphokinase
CNV	Copy number variant
CS	Calf serum
DMEM	Dulbecco’s modified Eagle’s medium
EMG	Electromyography
ER	Endoplasmic reticulum
FFAT	Two phenylalanines in an acidic tract
HGMD	Human Gene Mutation Database
IF	Immunofluorescence
MCS	Membrane contact sites
*MTTP*	Microsomal triglyceride transfer protein
*PANK2*	Pantothenate kinase 2
PCR	Polymerase chain reaction
PH-like domain	Pleckstrin homology-like domain
VAB	VPS13 adaptor binding
VAMP	Vesicle-associated membrane protein
VAP	VAMP-associated protein
VPS13A	Vacuolar protein sorting 13 homolog A
WB	Western blot
XK	X-linked Kx blood group antigen, Kell and VPS13A binding protein
