# Supplementary material for: A Novel VPS13A Deletion in VPS13A Disease (Chorea-Acanthocytosis): A Case Report with Brief Literature Summary
Source: Int J Mol Sci. 2025 Nov 27;26(23):11521. doi: 10.3390/ijms262311521 (PMC12692054; doi:10.3390/ijms262311521)
Supplement: Supplementary file 1 [file ijms-26-11521-s001.zip › ijms-3917049-supplementary.pdf]

Supplementary Table S1. *VPS13A* mutations related to ChAc phenotypes.

Variant (cDNA/Protein) NM_033305.3	Exon/ Intron	rsID	Mutation type	Classification (ClinVar/Varsome/ Franklin)	Clinical features (zygosity)	Population (n)	Reference
c.94_95insC p.W32S*13	Exon 1	N/A	Nonsense	-/LP/LP	22/M (cmpd het, c.130A>T): involuntary movements; OFIMS; involuntary movements of limbs; involuntary vocalizations; alopecia; ↑CK; acanthocytes.	Chinese (1)	[15]
c.130A>T p.K44*	Exon 2	N/A	Nonsense	-/-/LP	22/M (cmpd het, c.94_95insC): involuntary movements; OFIMS; involuntary movements of limbs; involuntary vocalizations; alopecia; ↑CK; acanthocytes.	Chinese (1)	[15]
c.144+1G>C	Intron 2	N/A	Nonsense	P/P/P	35/M (hom): involuntary movements; dys; dysphagia; dysarthria; neuropsychiatric symptoms; ↑CK; BCA and hyperintensity of caudate and putamen; acanthocytes.	Iranian (1)	[16]
c.145-2A>T	Intron 2	rs1447002141	Non-coding	P/P/P	30/F (cmpd het, c.4411C>T): involuntary movements of limbs, face, and tongue; epilepsy; CD; vocal tic; ↑CK; acanthocytes.<br><br>26/F (cmpd het, c.8211 +1232_8472-245delinsTC): atrophy of both caudate nuclei; epilepsy; OCD, alteration of personality, CD; ↑CK; acanthocytes.<br><br>35/F (cmpd het, c.2170+5G>A): involuntary movements of limbs and tongue; bilateral putamen and head of caudate nuclei atrophy; decreased muscle tone and power, bradykinesia, choreiform movement of limbs, neck, and tongue, ↑CK; acanthocytes.	Korean (3)	[17]<br>[18]<br>[19]
c.154-2A>G	Intron 2	N/A	Non-coding	-/LP/LP	26/M (hom): involuntary movements; OFIMS; involuntary movements of limbs; involuntary vocalizations; peripheral neuropathy; ↑CK; acanthocytes.	Chinese (2 brothers)	[15]

					31/F (hom): asymptomatic hyperCKemia; ↑CK.		
c.200T>C; p.L67P	Exon 4	rs1312425146	Missense	LP/-/LP	Reported in cmpd het with c.6098_6123del: clinical features not specified (details not reported separately for this variant).	American (1)	[20]
c.237delT; p.E80K*11	Exon 4	N/A	Nonsense	-/LP/LP	50/M (cmpd het, c.9429_9432del): TCS; speech difficulty; oromandibular dys; chorea generalized; impaired balance; hyperkinetic movements; dysarthria; hypophonia; tremor; ↑CK; caudate and lentiform nuclei atrophy; 25% acanthocytes.	British (1)	[21]
c.269T>A; p.I90K	Exon 4	rs119477052	Missense	-/P/VUS	Reported in cmpd het with c.6404_6405insT: clinical features not specified (details not reported separately for this variant).	American (1)	[22]
c.283+1G>A	Intron 4	N/A	Non-coding	-/LP/LP	18/M (cmpd het, c.9078-2A>G): seizures; memory impairment, disinhibition; muscle weakness; areflexia; polyneuropathic syndrome; ↑CK; 18% acanthocytes	German (1)	[23]
c.337C>T; p.Q113*	Exon 5	rs780664594	Nonsense	P/P/P	32/F (hom): involuntary UL movements; trunk spasms and gait disturbance; oromandibular dys; areflexia; hypotonia; bilateral and symmetrical striatal atrophy; 60% acanthocytes.<br><br>36/F (hom): seizures; psychiatric disturbances, depression and apathy; tremor of the right arm, akinesia, generalized hypotonia, DYS.	Moroccan (2 sisters)	[24]
c.495+1G>A	Exon 6	rs1447585760	Non-coding	P/LP/P	44/N/A (het): seizures; cognitive impairment; dys, dysphagia, vocal tics, OCD, gait instability, and peripheral neuropathy; ↑CK; 50% acanthocytes.<br><br>46/N/A (het): seizures; tremor, DYS, word-finding difficulties, memory impairment, impotence, and psychosis; ↑CK; 50% acanthocytes.	Canadian (2)	[25]
c.622C>T; p.R208*	Exon 9	rs1590003601	Nonsense	P/P-LP/P	30/M (hom): behavioural disturbance, slowness of daily activities, slowness in gait; involuntary movements of limbs, face, and tongue; cognitive impairment; apathy, depression; few acanthocytes.	Japanese (1)	[20]

c.755-1G>A	Intron 10	rs2131153683	Nonsense	P/P/P	29/M (hom): involuntary movements; dys; polymyopathy; ↑CK; BCA and hyperintensity of caudate and putamen; acanthocytes.	Iranian (1)	[16]
c.799C>T; p.R267*	Exon 11	N/A	Nonsense	P/P/P	44/F (hom): involuntary movements of limbs, face, and tongue; depression; ↑CK; atrophy of CS; acanthocytes (het).<br><br>42/F (hom): choreic movements; ↑CK; OCD and CD; atrophy of CS; acanthocytes.	Japanese (2)	[26] [18]
c.928C>T; p.Q310*	Exon 12	N/A	Nonsense	P/P/P	21/F (cmpd het, c.2427+316_5992-537dup): involuntary movements of limbs, face, and tongue; depression; ↑CK; atrophy of CS; acanthocytes.	Japanese (1)	[20]
c.994del; p.A332Lfs*10	Exon12	N/A	Nonsense	P/P/LP	Reported in cmpd het with c.6283del: clinical features not specified (details not reported separately for this variant).	Japanese (1)	[4]
c.1078C>T; p.Q360*	Exon 13	rs1447370088	Nonsense	-/P/P	11/M (cmpd het, c.7867C>T): tics, seizures; choreic movements; facial dyskinesia, DYS; LL-girdle; paroxystic atrial fibrillation; ↑CK; ~16% acanthocytes.<br><br>20/M (cmpd het, c.7867C>T): psychosis, OCD; choreic movements; dys, bradykinesia, postural tremor; sensory axonal polyneuropathy; LL-girdle; cardiac anomalies; ↑CK; 28% acanthocytes.	Italian (2 brothers)	[27]
c.1114_1115del; p.K372Vfs*4	Exon13	rs1353432136	Nonsense	P/P/P	49/F (hom): involuntary movements of limbs, face, and tongue; atrophy of CS; acanthocytes.<br><br>6/M (cmpd het, c.3817C>T): behavioural disorder; seizures; choreic movements; feeding dysphagia; psychiatric symptoms; atrophy of CN and hippocampus; sensori-motor polyneuropathy; chronic denervation with myopathy; LL girdle; ↑CK; ~15% acanthocytes.<br><br>24/M (cmpd het, c.3817C>T): anxiety disorder; akathisia and tics; psychiatric symptoms; sensor polyneuropathy; ↑CK; ~8% acanthocytes.	Italian (3; 2 brothers)	[20] [27]

c.1115del; p.K372Sfs*2	Exon 13	rs770350986	Nonsense	-/P/LP	Reported in hom with no more information: clinical features not specified (details not reported separately for this variant).	Italian (1)	[22]
c.1125_1128del; p.S375Rfs*23	Exon 13	rs748999076	Nonsense	P/P/P	Reported in het with no more information: clinical features not specified (details not reported separately for this variant).	Japanese (1)	[4]
c.1208_1211del; p.Q403Rfs*6	Exon 14	N/A	Nonsense	-/LP/LP	42/F (cmpd het, c.7867C>T): vocal tics; right-food dys; seizures choreiform movements; orofacial and tongue-dys; tremor, rigidity, bradykinesia; caudate and putamen atrophy; 70% acanthocytes.	British (1)	[21]
c.1287del; p.W430Gfs*29	Exon 13	N/A	Nonsense	P/P/P	18/M (cmpd het, c.4411C>T): seizures and alteration of personality.	Japanese (1)	[20]
c.1305G>A; p.W435*	Exon 15	N/A	Nonsense	-/-/LP	25/M (cmpd het, c.8035G>A): seizures; orofacial involuntary movements; choreic movements; dementia; atrophy of CS; acanthocytes.	Japanese (1)	[20]
c.1592delT; p.I531Kfs*7	Exon 17	rs1823633708	Nonsense	-/-/LP	Reported in hom with no more information: clinical features not specified (details not reported separately for this variant).	French (1)	[22]
c.1616C>G; p.S539*	Intron 20	rs758612012	Non-coding	P/LP/LP	Reported in het state with no more information: clinical features not specified (details not reported separately for this variant).	Japanese (1)	[4]
c.2029_2031del; p.H677del	Exon 20	N/A	Nonsense	-/VUS/VUS	Reported in cmpd het with c.9430_9432del: clinical features not specified.	Japanese (1)	[4]
c.2037+2T>G	Intron 20	N/A	Non-coding	-/-/LP	33/M (cmpd het, c.4411C>T): involuntary movements of limbs, face, and tongue; ↑CK; atrophy of CS; acanthocytes.	Japanese (1)	[20]
c.2134C>T; p.Q712*	Exon 21	N/A	Nonsense	-/LP/LP	22/M (cmpd het, c.6059del): involuntary movements of limbs, face, and tongue; OCD; ↑CK; atrophy of CS; acanthocytes.	Polish (1)	[20]
c.2191C>T; p.R731*	Exon 22	rs771943305	Nonsense	-/-/LP	33/M (cmpd het, c.3995_3996delinsA): involuntary movements of face, and tongue; mild dementia; ↑CK; atrophy of CS; acanthocytes.	Slovak (1)	[20]
c.2343del; p.K781Nfs*8	Exon 23	rs1488814919	Nonsense	P/P/P	N/A (hom)<br>18/F (hom): seizures; hyperkinetic movements; dysarthria; cognitive and psychiatric symptoms; mild caudate atrophy; acanthocytes.	Israelian (1)<br>Tunisian-Jewish (9)	[20]<br>[28]

					32/M (hom): seizures; hyperkinetic movements; dysarthria; cognitive and psychiatric symptoms; mild caudate atrophy; acanthocytes.<br><br>41/F (hom): seizures; hyperkinetic movements; dysarthria; cognitive and psychiatric symptoms; neuropathy.<br><br>29/F (hom): seizures; hyperkinetic movements; dysarthria.<br><br>16/F (hom): seizures; hyperkinetic movements; dysarthria; cognitive symptoms; mild cerebral atrophy; ↑CK; acanthocytes.<br><br>28/M (hom): seizures; hyperkinetic movements; mild caudate atrophy; ↑ CK; acanthocytes.<br><br>32/M (hom): seizures; hyperkinetic movements; ↑CK.<br><br>20/M (hom): tics and behaviour; hyperkinetic movements; cognitive symptoms; ↑CK; acanthocytes.<br><br>22/F (hom): seizures; hyperkinetic movements; dysarthria; cognitive symptoms; acanthocytes.		
c.2288_2502del	Exon 23	N/A	Large deletion	-/-/-	Reported in hom with no more information: clinical features not specified (details not reported separately for this variant).	Japanese (1)	[4]
c.2428-4036_2749delinsCATCCATTTAATT	Exons 24_26	N/A	Large deletion	-/-/LP	30/M (cmpd het, c.8211_1232_8472-245delinsTC): seizures; involuntary movements of limbs; depression and dementia; ↑CK.	Brazilian (1)	[20]
c.2427+316_5992-537dup	Exons 24_25	N/A	Large deletion	N/A	21/F (cmpd het with c.928C>T): seizures; OFIMS; depression; ↑CK; atrophy of CS; acanthocytes.	Japanese (1)	[20]
c.2512+1G>A	Intron 24	rs1488814919	Non-coding	-/P/P	42/M (hom): seizures; phonic tics; eating dysphagia; dysarthria; choreiform movements;	Iranian (1)	[16]

					axon/al sensory-motor polyneuropathy; caudate atrophy; ↑CK; acanthocytes.		
c.2593C>T; p.R865*	Exon 25	rs766404788	Non-coding	P/P/P	N/A (hom)<br><br>35/M (cmpd het, c.4411C>T): choreic movements; disinhibition and executive function disorder; OCD; ↑CK; atrophy of CS; acanthocytes.	Japanese (2)	[4]<br>[18]
c.2654_3122del	Exons 25_31	N/A	Large deletion	-/-/-	29/M (cmpd het, c. 8215G>T): involuntary movements of the lips, tongue, and limbs; uncontrolled frequent tongue and lip biting; dys; dyskinesia; ↑CK; 6% acanthocytes.	Chinese (1)	[29]
c.2824+1G>T	Intron 26	N/A	Non-coding	-/-/LP	18/F (cmpd het, c.8325G>A): choreic movements; seizures; delusion, OCD, disinhibition and CD; ↑CK; atrophy of CS; acanthocytes.<br><br>18/F (cmpd het, c.8325G>A): choreic movements; seizures; emotional instability, hallucination, frontal lobe dysfunction, OCD; ↑CK; atrophy of CS; acanthocytes.	Japanese (2)	[18]
c.2825-10T>G	Intron 26	N/A	Non-coding	-/VUS/VUS	28/M (cmpd het, c.7736_7739del); mood and behavioural disorders; bradykinesia, tremor, motor slowing, dys; atrophy of CN; LL-girdle; ↑CK.	Italian (1)	[27]
c.2833_2834delAA; p.K945Efs*11	Exon 26	N/A	Nonsense	P/VUS/LP	Reported in het state with no more information: clinical features not specified (details not reported separately for this variant).	Italian (1)	[22]
c.2951T>A; p.L984*	Exon 27	N/A	Nonsense	-/LP/LP	36/M (hom): TCS; involuntary movements of limbs, face, and tongue; axon/al sensory-motor polyneuropathy; dys; dysphagia; dysarthria; neuropsychiatric symptoms; ↑CK; BCA and hyperintensity of caudate and putamen; acanthocytes.	Iranian (1)	[16]
c.3109A>T; p.K1037*	Intron 28	N/A	Nonsense	P/P/LP	Reported in cmpd het with c.9474G>A: clinical features not specified (details not reported separately for this variant).	Japanese (1)	[4]
c.3157C>T; p.Q1053*	Exon 29	N/A	Nonsense	-/VUS/LP	N/A (hom)	Japanese (1)	[4]

c.3283G>C; p.A1095P	Exon 30	N/A	Missense	P/P/P	Reported in cmpd het with c.4835del: clinical features not specified (details not reported separately for this variant).	Japanese (1)	[4]
c.3419_3420del; p.T1140Rfs*6	Exon 32	N/A	Nonsense	-/P/VUS	35/M (cmpd het, c.3970_3973delAGTC): involuntary movements of limbs, face, and tongue; seizure; dementia; ↑CK; atrophy of CS; acanthocytes.	Japanese (1)	[30]
c.3556_3557dupAC ; p.V1187Lfs*12	Exon 33	rs779746050	Nonsense	P/P/P	32/F (hom): balancing problems; involuntary movements of the trunk, neck, and UL; compulsive lip and tongue biting; anxiety and depression; dysphagia, dysarthria, and orofacial dyskinesia; hypotonia of the legs and weight loss; ↑CK; acanthocytes 40%.<br><br>54/F (hom): multiple motor and verbal tics; paranoid behaviour; coprolalia; lip and tongue biting with buccolingual self-mutilation.	Mexican (2 sisters)	[31]
c.3562C>T; p.Q1188*	Exon 33	rs1312264385	Nonsense	P/-/P	23/M (cmpd het, c.4115-459_5991+6444dup): seizures; choreic movements; OFIMS; irritability and CD; ↑CK; atrophy of CS; acanthocytes.<br><br>26/F (cmpd het, c.4115-459_5991+6444dup): seizures; choreic movements; OFIMS; CD; ↑CK; atrophy of CS; acanthocytes.	Japanese (2)	[18]
c.3817C>T; p.R1273*	Exon 34	rs756096368	Nonsense	P/P/P	6/M (cmpd het, c.1114_1115delAA): behavioural disorder; seizures; choreic movements; feeding dys; psychiatric symptoms; atrophy of CN and hippocampus; sensori-motor polyneuropathy; chronic denervation with myopathy; LL girdle; ↑ CK; ~15% acanthocytes.<br><br>24/M (cmpd het, c.1114_1115delAA): anxiety disorder; akathisia and tics; psychiatric symptoms; sensor polyneuropathy; ↑ CK; ~8% acanthocytes.	Italian (2 brothers)	[27]
c.3889C>T; p.R1297*	Exon 34	N/A	Nonsense	-/-/LP	26/F (hom): involuntary movements of limbs, face, and tongue; seizures; dementia; ↑ CK; atrophy of CS; acanthocytes.	Japanese (1)	[20]

c.3903G>A; p.W1301*	Exon 34	rs200280742	Nonsense	P/P/LP	Reported in hom state with no more information: clinical features not specified (details not reported separately for this variant).	Hungarian (1)	[32] (Hungarian articol)
c.3970_3973del; p.S1324Kfs*4	Exon 35	rs1475930869	Nonsense	-/-/LP	35/M (cmpd het, c.3419_3420del): orofacial involuntary movement; seizures; dementia; ↑ CK; atrophy of CS; acanthocytes.	Japanese (1)	[30]
c.3995_3996del; p.F1332*	Exon 35	rs1475930869	Small deletion	P/P/P	33/M (cmpd het, c.2191C>T): OFIMS; mild dementia; ↑ CK; atrophy of CS; acanthocytes.	Slovak (1)	[20]
c.4419_4420insA; p.G1474Rfs*7	Exon 38	N/A	Nonsense	-/LP/LP	Reported in cmpd het with c.622C>T: clinical features not specified (details not reported separately for this variant).	Italian (1)	[22]
c.4242+1G>T	Intron 36	rs754245019	Non-coding	-/P/P	38/M (hom): movement disorder; tics; choreic movements; dysarthria; psychiatric disorders; neuropathy; caudate atrophy.<br><br>41/F (hom): movement disorder; tics; choreic movements; dysarthria; dysphagia; neuropathy.<br><br>15/M (cmpd het, exons 70_73 del): seizures; movement disorder; tics; choreic movements; dysarthria; neuropathy; caudate atrophy.<br><br>27/M (cmpd het, deletion of exons 70_73): movement disorder; tics; choreic movements; dysarthria; neuropathy; caudate atrophy.	French Canadian (4 siblings)	[33]
c.4346del; p.S1449Ffs*5	Exon 37	N/A	Nonsense	-/-/LP	Reported in het state with no more information: clinical features not specified (details not reported separately for this variant).	Japanese (1)	[4]
c.4411C>T; p.R1471*	Exon 37	rs1193250444	Nonsense	P/P/P	30/F (hom): seizures; OFIMS; dementia; ↑CK; atrophy of CS; acanthocytes.<br><br>44/M (hom): OFIMS; atrophy of CS; acanthocytes.<br><br>28/F (hom): alteration of personality; ↑CK; atrophy of CS; acanthocytes.<br><br>31/F (hom): seizures; OFIMS; depression; ↑CK; atrophy of CS; acanthocytes.	Japanese (13)	[20] (7 Pt)<br>[18] (6 Pt)

					42/F (hom): seizures; OFIMS; dementia; ↑CK; atrophy of CS.<br><br>33/M (het): OFIMS; ↑CK; atrophy of CS; acanthocytes.<br><br>18/M (cmpd het): seizures; alteration of personality (cmpd with c.1287del).<br><br>25/F (hom): OFIMS s; insomnia; ↑CK; atrophy of CS; acanthocytes.<br><br>35/M (hom): choreic movements; disinhibition and executive function disorder; ↑CK; atrophy of CS; acanthocytes.<br><br>34/M (hom): choreic movements; gait disturbance; ↑ CK; atrophy of CS; acanthocytes.<br><br>39/M (hom): choreic movements; orofacial involuntary movement; CD; ↑CK; atrophy of CS; acanthocytes.<br><br>33/F (hom): seizures; lack of motivation, violence and CD; atrophy of CS; acanthocytes.<br><br>25/F (hom): seizures; choreic movements; monologue and CD; ↑CK; atrophy of CS; acanthocytes.		
c.4592del; p.V1531Efs*20	Exon 38	N/A	Nonsense	-/-/LP	34/N/A (hom): seizures; Involuntary movements of limbs; ↑CK; acanthocytes.	Switzerland (1)	[20]
c.4956+1G>A	Intron 40	rs1085307823	Non-coding	-/P/P	Reported in cmpd with c.622C>T: clinical features not specified (details not reported separately for this variant).	Japanese (1)	[4]
c.5698del; p.N1900Tfs*10	Exon 44	rs1484976416	Nonsense	-/-/LP	29/M (cmpd het, c.6404dupT): seizures; involuntary movements of limbs, face, and tongue; delusion; ↑CK; acanthocytes.	Australian (1)	[20]

c.5761C>T; p.R1921*	Exon 44	rs1369905878	Nonsense	P/P/P	28/F (hom): seizures; involuntary movements of limbs, face, and tongue; dementia; ↑CK; atrophy of CS; acanthocytes.	Norwegian (1)	[20]
c.5715del; p.K1905Nfs*5	Exon 44	N/A	Nonsense	-/-/LP	29/F (cmpd het, c.7156-2A>T): seizures; involuntary movements of limbs; dementia; ↑CK; acanthocytes.	Brazilian (1)	[20]
c.5881C>T; p.R1961*	Exon 45	rs1286714220	Nonsense	P/P/P	44/M (hom): bilateral microphthalmos; blindness of the right eye and poor visual acuity of the left eye; oral involuntary movements; choreic movements; ↑CK; bilateral caudate and putamen atrophy; ophthalmologic disorders; acanthocytes.<br><br>40/M (hom): retinal dialysis and cataract; unstable gait, and choreic movement in the mouth, neck, limbs and hands; cognitive impairment; ↑CK; bilateral caudate and putamen atrophy.<br><br>20s/F (hom): seizures; choreic movements; CD; ↑CK; atrophy of CS; acanthocytes.	Iranian (2)<br>Japanese (1)	[34]<br>[18]
c.6059del; p.P2020Lfs*9	Exon 46	rs781242821	Nonsense	P/P/P	22/M (cmpd het, c.2134C>T): Involuntary movements of limbs, face, and tongue; OCD; ↑ CK; atrophy of CS; acanthocytes.<br><br>14/M (het): seizures; reduced attention span, disinhibition, psychosis; peripheral neuropathy; ↑CK; ~ 30% acanthocytes.<br><br>23/M (het): seizures; muscle proximal and distal weakness and atrophy; ↑CK; ~ 30% acanthocytes.	Polish (1)<br>German (2 brothers)	[20]<br>[23]
c.6094C>T; p.R2032*	Exon 46	rs764376151	Nonsense	-/P/P	Reported in het state with no more information: clinical features not specified (details not reported separately for this variant).	N/A	[4]
c.6098_6123del; p.S2033*	Exon 47	rs1174858706	Nonsense	-/P/P	Reported in cmpd het with c.6098_6123del: clinical features not specified (details not reported separately for this variant).	American (1)	[20]
c.6283del; p.2094Yfs*9	Exon 47	rs1587612257	Nonsense	P/P/P	Reported in cmpd het with c. 994del: clinical features not specified.	Japanese (1)	[4]

c.6404_6405insT; p.S2136Kfs*2	Exon 48	rs951347128	Nonsense	P/P/P	29/M (cmpd het, c.269T>A): seizures; involuntary movements of limbs, face, and tongue; delusion; ↑CK; acanthocytes.	Australian (1)	[20]
c.6700C>T; p.R2234*	Exon 48	rs748922513	Nonsense	-/P/P	Reported in cmpd het with c.8954-2A>C: clinical features not specified.	American (1)	[22]
c.7156-2A>T	Intron 51	rs1587628060	Non-coding	LP/LP/P	29/F (cmpd het, c.5715del): seizures; involuntary movements of limbs; dementia; ↑ CK; acanthocytes.	Brazilian (1)	[20]
c.7339_7340insT; p.Y2447LfsTer5	Exon 53	rs1419127345	Small insertion	-/P/P	Reported in het state with no more information: clinical features not specified (details not reported separately for this variant).	Japanese (1)	[4]
c.7411C>T; p.Q2471*	Exon 53	rs2131541108	Nonsense	-/-/LP	29/M (hom): seizures; OFIMS; dementia; ↑CK; acanthocytes.	Japanese (1)	[20]
c.7675_7676del; p.K2559Afs*16	Exon 55	N/A	Nonsense	LP	33/M (hom): OFIMS; mood instability; atrophy of CS; ↑CK; acanthocytes.	Japanese (1)	[20]
c.7736_7739del; p.R2579Nfs*26	Exon 55	rs748828128	Nonsense	-/P/P	28/M (cmpd het, c.2825-10T>G): mood and behavioural disorders; bradykinesia, tremor, motor slowing, dys; atrophy of CN; LL-girdle; ↑CK.	Italian (1)	[27]
c.7867C>T; p.R2623*	Exon 56	rs1055609567	Nonsense	P/P/P	42/F (cmpd het, c.1208_1211del): vocal tics; right-food dys; seizures; choreic movements; orofacial and tongue- dys; tremor, rigidity, bradykinesia; caudate and putamen atrophy; acanthocytes 70%.<br><br>26/M (cmpd het with c.9109C>T): seizures; OFIMS; dementia; ↑ CK; CS atrophy; acanthocytes.	British (1)<br>American (1)	[21]<br>[20]
c.8035G>A; p.A2679T	Exon 57	rs1587653832	Missense	LP/P/VUS	25/M (cmpd het, c.1305G>A): seizures; orofacial involuntary movements; choreic movements; dementia; atrophy of CS; acanthocytes.	Japanese (1)	[20]
c.8190_8191dup; p.W2731V*3	Exon 59	N/A	Nonsense	-/-/LP	29/M (hom): seizures; progressive paresis; impulse control disorder; muscle weakness and atrophy; peripheral neuropathy; ↑CK; 41% acanthocytes.	German (1)	[23]
c.8215G>T; p.E2739*	Exon 60	N/A	Nonsense	-/-/LP	29/M (cpmd het, exons 25_31del): involuntary movements of the lips, tongue, and limbs; uncontrolled frequent tongue and lip biting; dys; dyskinesia; ↑CK; 6% acanthocytes.	Chinese (1)	[29]

c.8211+1232_8472-245delinsTC	Exons 60-61	N/A	Large deletion	-/-/VUS	40/M (cmpd het, c.9403C>T): choreic movements; tic and truncal dys; mild muscular atrophy and weakness in LLs; ↑CK; ~5,6 % acanthocytes; hypokinesis, dilatation of left ventricular cavity and interstitial fibrosis of left ventricle.<br><br>-/F (hom): seizures; choreic movements; OFIMS; dementia; ↑CK; atrophy of CS; acanthocytes.<br><br>-/F (hom): seizures; choreic movements; OFIMS; dementia; ↑CK; atrophy of CS; acanthocytes.<br><br>36/M (hom): seizures; choreic movements; orofacial involuntary movements; dementia; ↑CK; atrophy of CS; acanthocytes.<br><br>N/A (hom)<br><br>20/F (hom): choreic movements; OFIMS; ↑CK; atrophy of CS; acanthocytes.<br><br>29/F (hom): seizures; choreic movements; OFIMS; dementia; ↑CK; atrophy of CS; acanthocytes.<br><br>Reported in a male in cmpd het with c. 9403C>T: clinical features not specified (details not reported separately for this variant).<br><br>Reported in cmpd het with c.8848_8860del: clinical features not specified.	Japanese (8)<br>Brazilian (1)	[35] (1 Pt)<br>[20] (8 Pt)
c.8472-1G>A	Intron 61	N/A	Non-coding	-/P/LP	Reported in cmpd het with c.2288+2T>C: clinical features not specified.	Japanese (1)	[4]
c.8501_8511del; p.F2834Ffs*6	Exon 62	N/A	Nonsense	-/-/LP	30/M (cmpd het, c.2428-4036_2749delinsCATCCATTTAATT): seizures; involuntary movements of limbs; depression dementia; ↑CK.	Brazilian (1)	[20]
c.8653dupT; p.Y2885Lfs*2	Exon 63	rs775734065	Nonsense	-/P/P	25/F (cmpd het, c.2532dupT): OFIMS; choreic movements; coprolalia; ↑CK; atrophy of CS; acanthocytes.	Japanese (1)	[18]

c.8848_8860del; p.N2950Lfs*14	Exon 65	rs1832691674	Nonsense	P/P/P	Reported in cmpd het with c.8211+1232_8472-245delinsTC: clinical features not specified.	Japanese (1)	[20]
c.8954-2A>C	Intron 66	N/A	Non-coding	-/-/LP	Reported in cmpd het with c.6700C>T: clinical features not specified.	American (1)	[22]
c.9078-2A>G	Intron 67	N/A	Non-coding	-/-/LP	18/M (cmpd het, c.283+1G>A): seizures; memory impairment, disinhibition; muscle weakness; areflexia; polyneuropathic syndrome; ↑CK; 18% acanthocytes	German (1)	[23]
c.9109C>T; p.R3037*	Exon 68	rs199807227	Nonsense	-/P/	N/A (cmpd het, c.9286_9289dupTTTG): progressive neurological illness; choreic movements; neurogenic muscular atrophy; acanthocytes.	Mexican (1)	[22]
c.9263T > G; p.M3088R	Exon 69	N/A	Missense	LP/VUS/VUS	25/M (hom): seizures; involuntary movements of jaw, tongue and UL; cognitive impairment; apathy, depression; ↑CK; few acanthocytes.	Indian (1)	[26]
c.9190_9360del; p.V3064_K3133del	Exons 69-70	N/A	Large deletion	N/A	34/F (hom): cardiopathy; laryngospasms; amenorrhea; alopecia; recurrent bruising; psychiatric disorders; choreic movements, ↑CK.	Italian (1)	**Our report**
c.9276-2A>T	Intron 69	N/A	Non-coding	P/P/P	-/F (hom): Involuntary movements of face, tongue and UL; acanthocytes.	Switzerland (1)	[20]
c.9286_9289dupTTTG; p.Thr3098CysfsTer12	Exon 70	N/A	Small insertion	P/P/P	N/A (cmpd het, c.9109C>T): progressive neurological illness; choreic movements; neurogenic muscular atrophy; acanthocytes.	Mexican (1)	[22]
c.9403C>T; p.R3135*	Exon 71	rs148173878	Nonsense	P/P/P	40/M (cmpd het, c.8211+1232_8472-245delinsTC): choreic movements; tic and truncal dys; mild muscular atrophy and weakness in LLs; ↑CK; ~5,6 % acanthocytes; hypokinesis, dilatation of left ventricular cavity and interstitial fibrosis of left ventricle.	Japanese (1)	[35]
c.9429_9432del; p.R3143Sfs*5	Exon 71	rs1085307750	Nonsense	P/P/LP	50/M (cmpd het, c.237delT): TCS; speech difficulty; oromandibular dys; chorea generalized; impaired balance; hyperkinetic movements; dysarthria; hypophonia; tremor; ↑CK; caudate and lentiform nuclei atrophy; 25% acanthocytes.	British (1)	[21]
c.9431_9432del; p.E3144Vfs*6	Exon 71	rs1085307750	Nonsense	-/LP/LP	25/M (hom): seizures; motor and vocal tics; choreic movements; distal-LL atrophy; ↑CK, acanthocytes.	Caucasian (3 brothers)<br>Japanese (1)	[36]<br>[4]

					22/M (hom): vocal tics; ataxia; choreic movements; OFIMS; axonal neuropathy; ↑CK; acanthocytes.<br><br>29/M (hom): seizures; OFIMS; distal and proximal LL muscle atrophy; ↑ CK; acanthocytes.<br><br>Reported in cmpd het with c.2030_2032del: clinical features not specified.		
c.9361_9695del	Exon 70_72	N/A	Large deletion	-/-/-	Reported as del exons 70–73 in Dobson 2002 in hom state with no more information: clinical features not specified (details not reported separately for this variant).	Japanese (1)	[4]

VPS13A: *Vacuolar protein sorting 13 homolog A*; *rsID*: Reference SNP cluster ID; P: pathogenic; LP: likely pathogenic; N/A: not available; hom: homozygous; het: heterozygous; cmpd het: compound heterozygous; TCS: tonic-clonic seizures; BCA: bilateral caudate atrophy; dys: dysthonia; LL: lower limb; UL: upper limb; *CD*: cognitive decline; OCD: obsessive-compulsive disorder; OFIMS: orofacial involuntary movements; BCA: Bilateral caudate atrophy; CS: corpus striatum; ↑*CK*: elevated creatine kinase.
